# The influence of eight cognitive training regimes upon cognitive screening tool performance in post-stroke survivors: a network meta-analysis

**DOI:** 10.3389/fnagi.2024.1374546

**Published:** 2024-07-19

**Authors:** Liqin Zhou, Xiaofeng Huang, Jieyu Wang, Fengming Wang, Jihong Liu, Nanhai Liu

**Affiliations:** ^1^School of the First Clinical Medicine, Gannan Medical University, Ganzhou, Jiangxi, China; ^2^Teaching and Research Section of Clinical Nursing, School of Nursing, Gannan Medical University, Ganzhou, Jiangxi, China; ^3^Neurology Department, LuoYang DongFang People’s Hospital, Luoyang Henan, China; ^4^Jiangxi Branch of National Clinical Research Center for Geriatric Diseases, The First Affiliated Hospital of Gannan Medical University, Ganzhou, Jiangxi, China

**Keywords:** cognitive training, computer-based cognitive training, virtual reality cognitive training, Multi-component training, post-stroke cognitive impairment, Treatment

## Abstract

**Background:**

Traditional meta-analysis offers only direct comparative evidence. The optimal cognitive training for poststroke cognitive impairment (PSCI) remains largely undetermined.

**Objectives:**

This study aims to assess and compare the effectiveness of selected cognitive training methods for PSCI patients and to identify and rank the most effective intervention programs.

**Methods:**

Searches were conducted in PubMed, Embase, Cochrane Library, Web of science, China National Knowledge Infrastructure, China Science and Technology Journal Database, Wanfang Database, and China Biomedical Database for randomized controlled trials up to September 30, 2023. Two researchers independently performed literature screening, data extraction, and quality assessment. Network meta-analysis was utilized to synthesize the main findings. The primary outcome focused on the intervention’s impact on subjective cognitive function, with secondary outcomes including effects on activities of daily living, motor function, and functional independence. This study is registered with PROSPERO (CRD42023463282).

**Results:**

Fifty eligible randomized controlled trials were identified, revealing eight distinct interventions. These interventions collectively demonstrate efficacy in enhancing cognition. Traditional cognitive training significantly improves overall cognitive function, daily living function, motor function, and functional independence. In Loewenstein Occupational Therapy Cognitive Assessment, Barthel Index, Fugl-Meyer Assessment, and Functional Independence Measure scales, a combination of computer-based and traditional cognitive training outperformed the conventional control group MD = 29.97 (95%CI: 16.3, 44.2), MD = 18.67 (95%CI: 9.78, 27.45), MD = 28.76 (95%CI: 5.46, 51.79) and MD = 42.2 (95%CI: 5.25, 78.99). In the MMSE scale, virtual reality cognitive training combined with traditional training was most effective MD = 8.01 (95%CI: 3.6, 12.4). On the MoCA scale, the combination of exercise and cognitive training showed superior results MD = 6.68 (95%CI: 2.55, 10.78). Only the combined computer-based and traditional cognitive training, as well as traditional cognitive training alone, significantly enhanced functional independence, with no notable differences in other pairwise interventions.

**Conclusion:**

The network meta-analysis suggests that augmenting traditional training with other modalities may enhance overall effectiveness. Specifically, interventions incorporating computer-based cognitive training appear to surpass other methods in improving cognition, daily living function, motor skills, and functional independence. The findings of this network meta-analysis provide evidence-based guidance for clinical decision-making.

**Systematic Review Registration:**

https://www.crd.york.ac.uk/PROSPERO/, identifier in PROSPERO (CRD42023463282).

## 1 Introduction

According to the 2020 American Heart Association’s report on heart disease and stroke statistics, the prevalence of stroke in the United States in 2016 was 2.5%, representing 7 million Americans over the age of 20 affected by stroke, resulting in nearly 800,000 stroke events and approximately 150,000 deaths (GBD 2019 Diseases and Injuries Collaborators, 2020). With advances in public awareness and the development of emergency medical services, such as stroke centers, the prognosis for stroke patients has improved globally. However, post-stroke cognitive impairment (PSCI) remains prevalent. PSCI typically manifests within 3 to 6 months post-stroke, encompassing not only defects due to the stroke lesion site but also pre-existing impairments. It primarily affects advanced visual-spatial functions, attention, and executive skills (Rost et al., 2022). PSCI is a leading cause of mortality and disability following a stroke (El Husseini et al., 2023), posing significant health and socioeconomic burdens on individuals, families, and communities.

Cognitive impairment encompasses deficits in functions such as memory, language, visual-spatial abilities, executive functions, calculation, and understanding and judgment. When two or more cognitive domains are impaired, affecting daily or social functioning, it may be classified as dementia. Currently, there is no FDA-approved medication for mild cognitive impairment (MCI). The American Academy of Neurology’s updated practice guideline summary does not recommend drugs for MCI; instead, it advocates regular exercise (grade B) and cognitive intervention (grade C) (Petersen et al., 2018). Cholinesterase inhibitors and the glutamic acid N-methyl-D-aspartate receptor antagonist memantine are the primary approved pharmacological treatments for cognitive impairment (Hermann and Zerr, 2022). Given the limitations and safety concerns of drug therapies, non-pharmacological interventions are recommended as the first-line treatment for dementia. Current clinical studies on non-drug interventions include personalized nursing, physical exercise, massage therapy, cognitive training, transcranial direct current stimulation, transcranial magnetic stimulation, acupuncture, and memory therapy (Li et al., 2021). Cognitive training (CT) involves structured task-based exercises aimed at enhancing cognitive processing. Its primary goal is to improve or maintain cognitive abilities. CT encompasses traditional paper-and-pencil methods, computer-based cognitive training (CBCT), virtual reality cognitive training (VRCT), and multi-component training [e.g., exercise-combined cognitive training (EX-CT)). Research indicates that patients with cognitive impairment generally benefit from cognitive training (Bahar-Fuchs et al., 2019; Li et al., 2022; Papaioannou et al., 2022]. The National Institute on Aging in the United States has found that cognitive training and physical exercise can enhance specific cognitive functions in the elderly. The Finland’s Geriatric Intervention Study to Prevent Cognitive Impairment and Disability demonstrated that combined interventions in nutrition, exercise, cognitive training, and social activities effectively improve or maintain cognitive function in older adults (Oh and Rabins, 2019).

Currently, numerous clinical studies worldwide have investigated various cognitive training treatments for PSCI (Pang et al., 2018; Cho and Lee, 2019; Sun et al., 2022). Earlier systematic reviews and meta-analyses have documented the therapeutic impacts of specific cognitive training methods on PSCI patients. For instance, a systematic review and meta-analysis by Xinming Chen et al. concluded that VRCT significantly enhances cognitive rehabilitation and daily living function recovery in PSCI patients, based on 21 randomized controlled trials (Chen et al., 2022). Another meta-analysis by Ruifeng Sun et al. demonstrated that combined cognitive training and exercise interventions markedly improve executive function and working memory in PSCI patients (Sun et al., 2021). Although previous meta-analyses suggest that CBCT might be more beneficial for cognition than VRCT (Xiao et al., 2022), these findings remain tentative. The most effective cognitive training type for enhancing cognitive abilities and quality of life in PSCI patients is still undetermined, as no study has concurrently compared multiple cognitive training models. Consequently, healthcare professionals face challenges in prescribing the most effective cognitive interventions for their patients. Network meta-analysis, which integrates both direct and indirect evidence to simultaneously compare the efficacy of multiple interventions, can rank these interventions based on different outcomes, thus providing evidence-based data to support medical decision-making. Therefore, the primary goal of this study is to compare and rank the effects of various cognitive training methods on the cognition and quality of life of PSCI patients by synthesizing existing evidence through network meta-analysis.

## 2 Methods

This study adhered to the Preferred Reporting Items for Systematic Reviews and Meta-analysis (PRISMA) guidelines (Page et al., 2021) and the Cochrane Handbook for Systematic Reviews of Interventions. The network meta-analysis was pre-registered in PROSPERO (CRD42023463282).

### 2.1 Data sources and retrieval

The systematic literature search was conducted using PubMed, Embase, Cochrane Library, Web of science, China National Knowledge Infrastructure, China Science and Technology Journal Database, Wanfang Database, and the Chinese Biomedical Database, covering the period from each database’s inception to September 30,2023. Additionally, a secondary search was conducted through the references of pertinent studies and reviews. The detailed search strategies are outlined in Supplementary Appendix 1.

### 2.2 Inclusion criteria

The inclusion criteria were formulated based on the PICOS (population, interventions, controls, outcomes, study design) framework.

#### 2.2.1. Population

The study population comprised stroke patients with cognitive impairment who met established diagnostic criteria. Inclusion criteria included: ① Age ≥ 18 years. ② No significant cognitive dysfunction prior to stroke. ③ Ability to participate in neuropsychological assessments and various cognitive training programs.

#### 2.2.2 Intervention methods

Interventions examined in this study encompass traditional cognitive training, CBCT, VRCT, and multi-component cognitive training (including EX-CT), and multi-component cognitive training (including exercise combined with cognitive training). These interventions were applied either individually or in combination.

#### 2.2.3 Control

Control groups in this study included participants receiving only exercise, traditional rehabilitation therapy, or alternate forms of cognitive training.

#### 2.2.4 Results

The primary outcomes focused on cognitive function, encompassing overall cognitive function, visual-spatial and executive functions, naming, language abilities, memory, attention, abstract thinking, delayed recall, and orientation. Cognitive function was assessed using scales such as the Montreal cognitive assessment (MoCA), mini-mental state ex-amination (MMSE), and Loewenstein Occupational Therapy Cognitive Assessment (LOTCA).

Secondary outcomes included improvements in daily living abilities, motor function, and functional independence. The Modified Barthel Index (MBI) or Barthel Index (BI) assessed activities of daily living, the Fugl-Meyer Assessment (FMA) evaluated motor function, and the Functional Independence Measure (FIM) scale was used for functional independence assessment.

#### 2.2.5 Research design

The study design was restricted to randomized controlled trial.

### 2.3 Data selection and extraction

All retrieved literature was imported into EndNote X9. Duplicates were eliminated, and studies were preliminarily selected based on titles and abstracts. Subsequently, it is necessary to download and read the full texts to include primary studies that meet the research criteria. Before data extraction, an electronic information extraction form tailored to this project’s standards is used for evaluation. Two researchers (a and b) independently extracted data from the included studies, covering characteristics such as the first author’s name, year of publication, research location (country), citation, study type, participant characteristics, experimental and control group interventions, intervention duration, frequency, participant age, gender, and outcome measures. Discrepancies in literature screening and data extraction were resolved through discussion between the two researchers.

### 2.4 Literature quality evaluation

Two researchers independently assessed the risk of bias in all included studies using the Rob2.0 tool, as recommended in the Cochrane Handbook (Sterne et al., 2019). The assessment encompassed randomization methods, blinding, allocation concealment, intervention allocation, compliance, missing outcome data, outcome measurement, and selective outcome reporting. Each category was classified as low risk, unclear, or high risk. Any disagreements were discussed and resolved by the two researchers.

### 2.5 Statistical analysis

#### 2.5.1 Analysis methods

For network meta-analysis, RStudio 4.3.1 software with the gemtc package (version 1.0-1) and JAGS software were used, employing the Markov Chain Monte Carlo method within a Bayesian framework. Four Markov chains were implemented for simulation analysis, with an initial value of 2.5, a refinement iteration step of 1, 5000 pre-simulation iterations for annealing, and 20,000 iterations to achieve model convergence. The deviation information standard (DIC) was utilized to compare model fit and overall consistency (a DIC difference of less than five between consistency and inconsistency models indicated the use of a consistency model). In the presence of closed loops, the node-splitting method was applied to assess local consistency.

All dichotomous outcomes were expressed as risk ratio (RR), with the corresponding 95 % CI reported. A statistically significant difference was considered when the value (GBD 2019 Diseases and Injuries Collaborators, 2020) was not included in the 95% CI. The random effects model within a Bayesian framework was employed to simultaneously analyze the efficacy of all treatment regimens. The analysis results comprised network diagrams for each outcome index, cumulative probability ranking diagrams, league tables, and ‘correction-comparison’ funnel plots. The surface under the cumulative ranking curve (SUCRA) served as an index for cumulative ranking probability, ranking the interventions’ advantages and disadvantages based on the SUCRA value. The closer the value to 100%, the more effective the intervention was deemed. Pairwise and Network Meta-Analysis were conducted using Stata 15.1 and R software (VER.4.3.1).

## 3 Results

### 3.1 Baseline characteristics and quality of included studies

**FIGURE 1:**
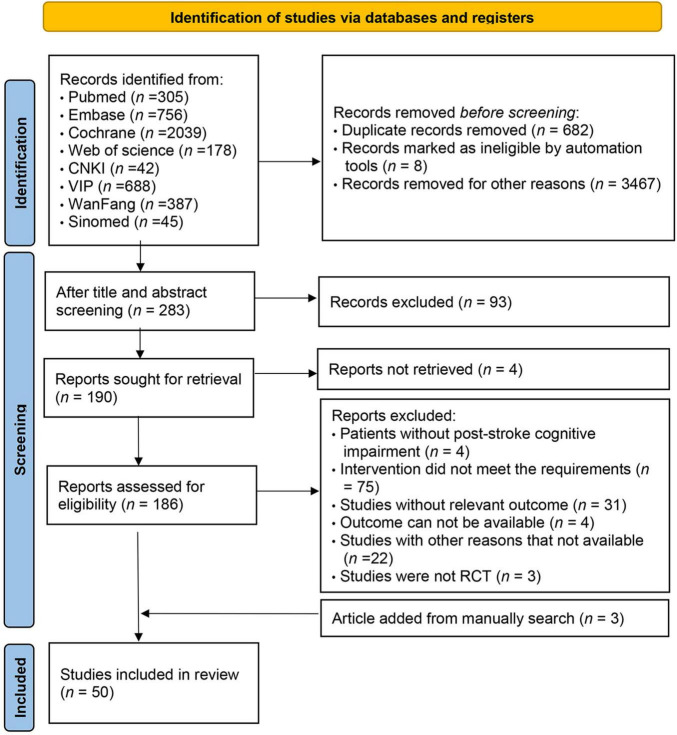
Literature screening flow chart.

Initially, 4440 studies were screened. After removing duplicates, 3758 articles were reviewed, with 3484 excluded based on title and abstract. Consequently, 283 articles were assessed in full text, adhering strictly to the inclusion and exclusion criteria. Ultimately, 47 studies met the inclusion criteria. Additional searches in the references of published systematic reviews identified three more eligible studies, resulting in 50 randomized controlled trials included (Chen et al., 2006, 2019, 2020; Liu et al., 2006, 2019, 2022; Ou et al., 2007; Shin et al., 2008; Yang and Liu, 2008; Wang et al., 2009, 2013; Li and Gan, 2010; Kim et al., 2011; Zhang et al., 2012, 2018, 2020; Feng et al., 2014; Ye et al., 2014; Zucchella et al., 2014; Xu et al., 2015; Yoo et al., 2015; Li M. et al., 2016; Li X. et al., 2016; Cao et al., 2017; Gong, 2017; Jiang et al., 2017; Li et al., 2017; Lv et al., 2017; Maier et al., 2017; De Luca et al., 2018; Prokopenko et al., 2018, 2019; Xiao, 2018; Mao et al., 2019; Wang, 2019; Xiao and Liang, 2019; Yeh et al., 2019, 2022; Faria et al., 2020; Fu et al., 2020; Jung et al., 2020; Shao, 2021; Xuefang et al., 2021; Ho et al., 2022; Liao et al., 2022; Shang et al., 2022; Sun et al., 2022; Xia et al., 2022; Zhao et al., 2020, 2022). The specific screening process is depicted in Figure 1. These studies encompassed 3063 participants, with an average sample size of 61 subjects (range 11–149) and an age range of 42–74 years. Of these, 1274 (41.6%) were female and 1789 (58.4%) male. The geographic distribution of the 50 trials included 74% from China, 6% from Taiwan, 6% from South Korea, 4% each from Italy and Russia, and 2% each from the United States, Portugal, and Spain. Attempts to collect racial data were largely unsuccessful due to infrequent reporting. Eleven different cognitive interventions were administered, including: (1) VRCT (*n* = 2). (2) VRCT combined with traditional cognitive training (*n* = 6). (3) VRCT combined with CBCT (n = 1). (4) CBCT (*n* = 17). (5) CBCT combined with traditional cognitive training (n = 9). (6) Cognitive training (*n* = 42). (7) Exercise combined with CBCT (*n* = 2). (8) Exercise combined with traditional cognitive training (*n* = 1). The baseline characteristics of the subjects are shown in Table 1.

**TABLE 1:** Baseline characteristic information table.

References	Study type	Sample size	Gender (male/fe-male)	Age Mean ± SD or Median (IQR) or Median [range] or range	Experimental intervention		Control		Cognitive domain targeted by training	Main Outcom-es
					Training (frequency/ duration)	detail	Training (frequency/ duration)	detail		
(Xiao and Liang, 2019) China	RCT	E: 16	E: 9/7	E: 67.74 ± 9.35	VRCT_CT (40min/d,5d/w,4w)	VRCT: Based on the Kinect somatosensory interaction technology (2 games, playing tennis and cutting fruit) CT: Memory training, attention training, computational ability training, training on spatial, structure and orientation function and Executive ability training	CT (40min/d,5d/w,4w)	Memory training, attention training, computational ability training, training on spatial structure and orientation function and executive ability training	Cognitive function and daily living abilities	MMSE MoCA MBI P300
		C: 18	C: 12/6	C:70.59 ± 10.67						
(Fu et al., 2020) China	RCT	E:18	E:10/8	E:42∼62 (53.61 ± 6.35)	VRCT_CT (45min/d,6d/w,4w)	VRCT: Based on the STB-110 99PALPAL machine produced by Man & tel (5 training modules, e.g. Count operation, fruit picking, jigsaw, Handicap runway and Photo pairing) CT: NA	CT (45min/d,6d/w,4w)	NA	Cognitive functions and balance functions	MMSE
		C:18	C:11/7	C:45∼67 (55.33 ± 6.54)						
(Xuefang et al., 2021) China	RCT	E:59	E:29/30	E:62.7 ± 1.6	VRCT_CT (120min/d,7d/w,3w)	VRCT: NA CT: Orientation training, attention Training, calculation training, memory training, language training and training the ability to solve problems	CT (120min/d,7d/w,3w)	Orientation training, attention Training, calculation training, memory training, language training and training the ability to solve problems	Attention and motor function	MoCA NIHSS NSE BDNF
		C:59	C:30/29	C:65.3 ± 1.4						
(Maier et al., 2017) Spain	RCT	E:6	E:4/2	E:66.33 (6.80)	VRCT_CT (30min/d,5d/w,6w)	VRCT: Rehabilitation Gaming System (RGS), (3 cognitive training scenarios, e.g. the Spheroids scenario, the Star Constellations scenario and the Quality Controller scenario) CT: 30 individual cognitive tasks (e.g. draw figures mirrored, complete sentences or word search puzzle etc.)	CT (30min/d,5d/w,6w)	30 individual cognitive tasks (e.g. draw figures mirrored, complete sentences or word search puzzle etc.)	Attention, memory and executive function	MMSE BI Fugl-Meyer FM-UE WAIS TMTA TMTB RAVLT
		C:5	C:2/3	C:64.00 (7.00)						
(Jung et al., 2020) America	RCT	E:14	E:2/12	E:72.71 ± 9.86	VRCT_CT (30min/d,2d/w, 12w)	VRCT: Based on the Neuro-World—a set of six serious games for cognitive training on mobile devices. CT: NA	CT (30min/d,2d/w,12w)	NA	Cognitive functions and working memory	MMSE DBS DFS GDS SUS
		C:15	C:2/13	C:72.67 ± 12.64						
Mao et al. (Mao et al., 2019) China	RCT	E:30	E:16/14	E:54.300 ± 7.28	VRCT _CT (VRCT 20min/d,5d/w,8w) + (CT 30min/d,5d/w,8w)	VRCT: Based on MNST V1.0 therapeutic instrument produced by Suzhou Minster Medical Technology Co. LTD (Nearly 300 action videos related to daily life, such as peeling peanuts and cutting fruit) CT: Schulte square method, onoception and tactile training, memory training for daily living activities and Assignment operation	CT (30min/d,5d/w,8w)	Schulte square method, onoception and tactile training, memory training for daily living activities and Assignment operation	Upper limb function and cognitive function	MoCA MBI Fugl-Meyer (FMA-UE) WCST
		C:30	C:15/15	C:56.867 ± 6.14						
(Liu et al., 2022) China	RCT	E:15	E:9/6	E:74.93 ± 6.81	VRCT (15min/d,6d/w,6w)	VRCT: Head-mounted displays (3 categories: life skills training, exergames and entertaining games) The difficulty level of each game is divided into five stars	CT (15min/d,6d/w,6w)	Processing speed and attention training, memory training, computational ability training, executive and problem-solving ability training	Global cognitive, episodic memory, verbal memory, attention, daily living ability, executive ability and spatial orientation	MoCA MBI DBS DFS TMTA
		C:15	C:8/7	C:73.40 ± 7.5						
(Faria et al., 2020) Portugal	RCT	E:14	E:5/9	E:59.14 ± 11.81	VRCT (45min/d,3d/w,8w)	Reh@City v2.0 (adaptive cognitive training through everyday tasks VR simulations)	CT (45min/d,3d/w,8w)	Task Generator (TG: content equivalent and adaptive paper-and-pencil training)	Attention, memory, executive functions and language specific domains	MoCA DST WAIS-III TMTA TMTB
		C:18	C:11/7	C:65.00 ± 6.20						
(Kim et al., 2011) Korea	RCT	E:15	E:5/10	E:66.5 ± 11.0	VRCT-CBCT (VRCT30min/d, 3d/w,4w) + (CBCT30min/d, 5d/w)	VRCT: Virtual reality system and IREX system^®^ CBCT:Computer-assisted cognitive rehabilitation used Com Cog^®^	CBCT (30min/d,5d/w,4w)	Computer-assisted cognitive rehabilitation used Com Cog^®^	Attention, memory, basic visual perception and auditory perception	K-MMSE K-MBI TOL DST
		C:13	C:6/7	C:62.0 ± 15.8						
(Shang et al., 2022) China	RCT	E:39	E:23/16	E:58.89 ± 10.58	CBCT_CT (25-45min/d,6d/w,4w) + (35-45min/d,6d/ w,4w)	CBCT: Based on v1.0 version of the language cognitive system for computer-assisted cognitive training (memory ability training, attention ability training, reasoning ability training, computational ability training, directional ability training and language ability training) CT: Memory ability training, attention ability training, computational ability training and directional ability training	CT (35-45min/d,6d/w,4w)	Memory ability training, attention ability training, computational ability training and directional ability training	Global cognitive function	MoCA
		C:38	C:21/17	C:59.23 ± 5.98						
Liu et al. (2019) China	RCT	E:15	E:9/6	E:58.33 ± 8.19	CBCT_CT (30min/d, 5d/w,12w)	CBCT: Based on the sixth brain rehabilitation system’s computer-assisted cognitive training (memory, attention, sensory perception, agility, executive ability, computation and reasoning training) CT: Memory training, attention training, visual space and executive function training, computational force training and directional force training	CT (30min/d,5d/w,12w)	Memory training, attention training, visual space and executive function training, computational force training and directional force training	Global cognitive function	MoCA MBI FIM
		C:15	C:7/8	C:54.40 ± 9.23						
(Chen et al., 2019) China	RCT	E:24	E:14/10	E:60.46 ± 7.51	CBCT_CT (CBCT30min/ d,6d/w,6w) + (CT30min/ d,6d/w,6w)	CBCT: Based on computer cognitive impairment diagnosis and treatment system (ZM 3.2 system) developed by Jinan University (attention specific training, continuous attention function training, selective attention function training, metastatic attention function training and attention and coordination operation training) CT: NA	CT (30min/d,6d/w,6w)	NA	Attention function and global cognitive function	MoCA MBI
		C:24	C:15/9	C:59.96 ± 7.68						
(De Luca et al., 2018) Italy	RCT	E:20	E:11/9	E:43.9 ± 16.6	CBCT_CT (CT45min/ d,6d/w,8w)+ (CBCT45min/ d,3d/w,8w)	CBCT: A neurocognitive-rehabilitative training consisting of 24 sessions of BTsN CT: A standard cognitive rehabilitation training	CT (45min/d,6d/w,8w)	A standard cognitive rehabilitation training	Trunk control, visuo-spatial and constructive abilities	MMSE TMTA TMTB HRS-A HRS-B
		C:15	C:7/8	C:42.1 ± 17.7						
(Wang et al., 2013) China	RCT	E:24	E:14/10	E:59.7 ± 3.9	CBCT_CT (CT30min/ d,5d/w,8w)+ (CBCT30min/ d,5d/w,8w)	CBCT: Based on The German Re-hacom Cognitive Rehabilitation System (reaction behavior, space operation capability, plane operation and attention, figure memory, logical thinking, computer power, moving eyes and search capability) CT: Orientation training, memory training, computational training and logical thinking ability training	CT (CT30min/d,5d/w,8w)	Orientation training, memory training, computational training and logical thinking ability training	Global cognitive function	MoCA LOTCA
		C:24	C:12/12	C:59.5 ± 6.2						
(Wang, 2019) China	RCT	E1:15	E1:11/4	E1:62.3 ± 5.2	E1: CBCT_CT E2: CT E3: CBCT (30min/d,5d/w,8w)	CBCT: Based on the Taiyige Cognitive Training System CT: Visual and spatial structure ability training, executive function, problem solving ability training, attention training, memory training and computing power training	Control (30min/d,5d/w,8w)	Conventional rehabilitation treatment (comprehensive training, exercise therapy and occupational treatment for patients with limb dysfunction)	Global cognitive function	MoCA BI LOTCA
		E2:15	E2:11/4	E2:62.3 ± 5.2						
		E315	E3:11/4	E3:62.3 ± 5.2						
		C:15	C:11/4	C:62.3 ± 5.2						
(Li M. et al., 2016) China	RCT	E:35	E:21/14	E:47.5 ± 4.8	CBCT_CT (CT35-4min/ d,6d/w)+ (CBCT30min/d,6d/w)	CBCT: Based on computer-assisted cognitive rehabilitation system training (space operation ability training, attention and plane operation ability training and eye training) CT: Thinking ability training, language communication training, computational ability training, attention training and directional ability training	CT (35-45min/d,6d/w)	Thinking ability training, language communication training, computational ability training, attention training and directional ability training	Global cognitive function	MMSE LOTCA
		C:35	C:20/15	C:47.3 ± 4.6						
(Cao et al., 2017) China	RCT	E:25	E:16/9	E:56.96 ± 12.50	CBCT_CT (CT30min/ d,5d/w,8w)+ (CBCT30min/ d,5d/w,8w)	CBCT: Based on Dr.Brain-2 Dr. Qihui Cognitive ability test and training system (Junior high school stage: attention training, observation training, memory training, digital cognitive training, figure cognition training, sequence cognitive training, alien identification, same type matching. advanced cognition: digital reasoning, situational cognition, graph reasoning, logical analogy, alien identification, network reasoning semantic understanding, coordinate reasoning memory strategy problem solving) CT: Orientation training, memory training, calculation and logical thinking training	CT (30min/d,5d/w,8w)	Orientation training, memory training, calculation and logical thinking training	Global cognitive function	MoCA MMSE
		C:25	C:13/12	C:57.12 ± 13.31						
(Zhang et al., 2020) China	RCT	E:37	E:20/17	E:71.5 ± 5.3	CBCT_CT (20min/ d,7d/w,12w) + (30min/ d,5d/w,12w)	CBCT: Ask the patient to correctly click on the moving object in the screen, let the patient remember the pre-designed graphics, and then memorize them together according to the size and shape of the model CT: Language, attention, and thinking ability training	CT (30min/d,5d/w,12w)	Language, attention, and thinking ability training	Reactivity ability, attention, and plane manipulation ability	MMSE QOL HDS
		C:37	C:21/16	C:71.6 ± 5.7						
(Zhao et al., 2020) China	RCT	E:77	E:53/24	E:59.58 ± 11.53	CBCT (30min/d,5d/w,2w)	All engaging small games	CT (30min/d,5d/w,2w)	Visual space and executive function, naming, memory, attention, language, abstract, delayed memory, and orientation	Instant memory, computational ability, and delayed memory cognitive ability	MoCA MMSE
		C:72	C:48/24	C:62.63 ± 13.68						
(Lv et al., 2017) China	RCT	E:42	E:31/11	E:67.55 ± 8.62	CBCT (60min/d,7d/w,2m)	Based on computer-assisted cognitive rehabilitation system training (computer attention, memory, computation power, thinking and perception of five aspects of systematic training).	CT (60min/d,7d/w,2m)	Memory training, attention training, computational power training and daily thinking ability discrimination training anonymous training	Cognitive function, motor function and activities of daily living	MMSE FIM Fugl-Meyer
		C:40	C:27/13	C:68.12 ± 8.59						
Jiang et al. (2017) China	RCT	E:25	E:13/12	E:61.32 ± 16.83	CBCT (30-40min/d,5d/w,4w)	Based on cognitive impairment diagnosis and treatment device ZM3.1 training system (orientation ability, focus ability, structure ability, computing ability, memory ability, reasoning ability, language ability and other training modules)	CT (30-40min/d,5d/w,4w)	Orientation ability, retelling ability, calculation ability, memory ability, recognition ability, understanding ability, expression ability and structure imitation ability	Time oriented force and computing power	MMSE
		C:25	C:14/11	C:65.28 ± 13.03						
(Shao, 2021) China	RCT	E:54	E:29/25	E:57.46 ± 6.448	CBCT (30min/d,5d/w,8w)	Based on computer-aided cognitive training system (attention training, memory training, training of visual spatial ability, language ability training, training of computational force and training of executive force)	CT (30min/d,5d/w,8w)	Attention, computing power, memory, execution, orientation and other aspects of the training	Cognitive function	MoCA MMSE
		C:48	C:22/26	C:58.88 ± 6.822						
(Xu et al., 2015) China	RCT	E:36	E:20/16	E:60.65 ± 15.20	CBCT (30min/d,6d/w,8w)	Based on computer software (orientation, visual perception, spatial perception, action application, visual movement organization, thinking action)	CT (30min/d,6d/w,8w)	Using images and daily necessities to provide one-on-one cognitive training (orientation, visual perception, spatial perception, action application, visual movement organization and thinking action)	Cognitive function	MMSE LOTCA
		C:36	C:22/14	C:56.71 ± 14.90						
(Xiao, 2018) China	RCT	E:30	E:22/8	E:56.10 ± 13.23	CBCT (30min/d,5d/w,4w)	Self-developed cognitive rehabilitation training system based on information processing theory (sensory and perceptual training, attention and memory training)	CT (30min/d,5d/w,4w)	Attention training, memory training, computational power training, identification training and anonymous training	Cognitive function, attention, memory, and executive functions	MOCA MBI LADL TMTA TMTB DBS DFS
		C:30	C:23/7	C:57.90 ± 9.47						
(Liao et al., 2022) China	RCT	E:30	E:13/17	E:58.30 ± 6.12	CBCT (30min/d,5d/ w,12w)	Based on the sixth brain rehabilitation system’s computer-assisted cognitive training (execution function training, memory training, attention training, visual spatial structure ability training, computational power training, logical thinking training, and language ability training)	CT (30min/d,5d/w,12w)	Memory training, attention training, visual spatial execution ability training, and directional ability training	Cognitive function	MoCA MMSE
		C:30	C:18/12	C:60.16 ± 5.12						
(Ho et al., 2022) Taiwan, China	RCT	E:19	E:12/7	E:63.63 (11.27)	CBCT (20min/d,2d/ w,12w)	Administered through a touch-screen computer equipped with Lumosity software (suitable difficulty level).	CT (20min/d,2d/w,12w)	Involving paper-and-pencil tasks and tabletop tasks, such as board games, puzzles, card games and memory games.	Information processing speed, attention and memory	MMSE ADL DST SDMT
		C:20	C:14/6	C:65.50 (8.28)						
(Prokopenko et al., 2019) Russia	RCT	E:23	E:13/10	E:59[54.9;66.5]	CBCT (30-40min/d,10d)	The authors’ set of original computerized stimulation programs	Control (10d)	Standard rehabilitation courses	Visuospatial memory, attention, visual memory and count	MoCA MMSE FAB HADS NIHSS
		C:26	C:19/7	C:60.5[55.8;68.8]						
(Prokopenko et al., 2018) Russia	RCT	E:10	E:6/4	E:59.5 [57; 60]	CBCT (30-40min/d,10d)	The original complex of neuropsychological programs developed at Krasnoyarsk State Medical University (krassmu)	Control (10d)	Standard rehabilitation courses	Memory, attention, counting etc.	MMSE FAB LADL HADS_A HADS-D NIHSS
		C:9	C:8/1	C:62.55 [61; 65]						
(Ye et al., 2014) China	RCT	E:30	E:21/9	E:60.33 ± 9.60	CBCT (30min/d,5d/w,8w)	Based on the corresponding cognitive rehabilitation module of the RehaCom Cognitive Training System (modified version) in Germany (reaction behavior training; spatial operation ability training; plane recognition ability training; graphic memory training; logical thinking ability training; calculation ability training; eye movement training; search ability training and attention training)	Control (8w)	Conventional internal medicine treatment and routine rehabilitation training.	Global cognitive function	MoCA MMSE FIM LOTCA
		C:30	C:23/7	C:60.33 ± 9.60						
(Xia et al., 2022) China	RCT	E:32	E:18/14	E:56.76 ± 1.85	CBCT	Sensory and perceptual training, Attention and memory training, complex cognitive functions	Control	NA	Global cognitive function	MoCA
		C:32	C:16/16	C:55.35 ± 2.12						
(Shin et al., 2008) Korea	RCT	E:33	E:18/15	E:60.6 ± 17.5	CBCT (30min/d,5d/w,4w)	RehaCom software consisted of reaction behavior, memory of words and topological memory programs.	Control (4w)	Conventional rehabilitation therapy including physical and occupational therapy	Global cognitive function	MMSE FIM CNI LOTCA
		C:24	C:16/8	C:59.1 ± 18.6						
(Yoo et al., 2015) Korea	RCT	E:23	E:8/15	E:53.2 ± 8.8	CBCT (30min/d,5d/w,5w)	The RehaCom software	Control (5w)	Rehabilitation therapy including physical and occupational therapy.	Cognitive function, digit span, visual span, visual learning and activities of daily living	FIM DST TMT CNT
		C:23	C:9/14	C:56.3 ± 7.9						
(Zhang et al., 2012) China	RCT	E:40	E:25/15	E:61.41 ± 5.65	CT	Memory training, training of attention and coordination, logic thinking training, computational force training and the perceived impairment training	Control	NA	Cognitive function and activities of daily living	MMSE MBI
		C:40	C:23/17	C:65.25 ± 4.06						
(Li et al., 2017) 2017 China	RCT	E:46	E:24/22	E:55.1 ± 10,6	CT (60min/d,5d/w,2m)	Filling in graphics, piecing together graphics, classifying physical images, arranging story images, and simulating outdoor shopping	Control (2m)	NA	Cognitive function	MoCA FMA
		C:50	C:26/24	C:56.1 ± 11.2						
(Li X. et al., 2016) China	RCT	E:51	E:28/25	E:63.9 ± 8.3	CT (50min/d,7d/ w,15d)	Attention training (relax, sign language song, rhythmic, description the picture, medical schulte square for training, anti-interference training, domino card practice, identify pictures, identify changes in objects and people, finger exercises)	Control (15d)	Rehabilitation training includes guiding patients on chart,15dreading and literacy training	Attention	MoCA
		C:51	C:25/24	C:63.9 ± 8.3						
(Feng et al., 2014) China	RCT	E:30	E:21/9	E:63.37 ± 7.65	CT (90min/d,3d/w,6w +120min/d,3d/w, 6w)	Memory training, attention training, visuospatial structure ability training, computation ability training, executive function and problem-solving ability training, orientation ability training and language and communication ability training	Control (12w)	Routine nursing education and routine rehabilitation training	Cognitive function and activities of daily living	MoCA BI NIHSS
		C:30	C:22/8	C:62.01 ± 7.81						
(Liu et al., 2006) China	RCT	E:46	E:28/18	E:66.7 ± 4.1	CT	Attention training, directional force training, visuospatial structure ability training, memory training, computational power training, executive function and problem solving ability training and speech and communication ability training	Control	NA	Cognitive function, motor function and activities of daily living	NCSE MBI
		C:46	C:29/17	C:67.2 ± 3.2						
(Chen et al., 2020) China	RCT	E:23	E:14/9	E:51 ± 19	CT (30-45min/d,5d/w)	Attention training, memory training, computational training, thinking training, and perceptual training	Control (6w)	Rood technology, brunnstrom technology, and MRP exercise relearning technology	Cognitive function, limb function and independence of daily life	FMA FIM LOTCA
		C:23	C:16/7	C:58 ± 17						
(Chen et al., 2006) China	RCT	E:30	E:16/14	E:62.12 ± 6.98	CT (>100min/d, 5d/w,5-8w)	Attention training, memory training, computational training, thinking and reasoning training, and relevant corrective treatment for patients with amnesia and apraxia	Control (>100min/d,5d/w,5-8w)	NA	Cognitive function	LOTCA FMA FIM
		C:30	C:18/12	C:63.60 ± 7.43						
(Ou et al., 2007) China	RCT	E:28	E:20/8	E:61.93 ± 6.90	CT (30min/d,6d/w,5w)	Attention training, memory training, computational training, and cognitive reasoning training	Control (5w)	Neurodevelopmental therapy and daily living ability exercises	Cognitive function, motor function and activities of daily living	NCSE FMA BI
		C:30	C:19/11	C:62.20 ± 7.32						
(Zucchella et al., 2014) Italy	RCT	E:42	E:23/19	E:64 [56.2;74.2]	CT (60min/d,4d/w,4w)	Included 45 minutes of therapist-guided computer exercises using two software programs: “Una palestra per la mente” and “Training di riabilitazione cognitiva”	Control (4w)	Spent the same amount of time with a psychologist, discussing general topics, news and their recent activities	Time orientation, spatial orientation, visual attention, logical reasoning, memory and Executive function	MMSE Digit Span TMTA TMTB FIM
		C:45	C:23/22	C:70 [62.5;76.5]						
(Zhao et al., 2020) China	RCT	E:30	E:17/13	E:60.72 ± 1.28	CT (45min/d,5d/w,4w)	Memory training, daily living ability training, calculating power and writing training and directional force training	Control (4w)	General balance ability, body activity ability, gait ability, concentration, daily life training	Cognitive function	MoCA MMSE BI
		C:30	C:18/12	C:60.62 ± 1.38						
(Zhang et al., 2018) China	RCT	E:85	E:49/36	E:50.5 ± 10.2	CT (45min/d,5d/w,4w)	Spatial orientation, sensational function, attention, thinking function and directive force	Control (4w)	Psychological care, health education and life ability training	Cognitive function and activities of daily living	MoCA MBI
		C:85	C:47/38	C:50.3 ± 10.4						
(Li and Gan, 2010) China	RCT	E:10	E:6/4	E:71.0	CT	Memory rehabilitation training, language function training, understanding training and problem solving ability training	Control (4w)	NA	Cognitive function	MMSE
		C:10	C:7/3	C:73.2						
(Yang and Liu, 2008) China	RCT	E:18	E:10/8	E:62 ± 4	CT (60 min/d,4d/w,4w)	Using card methods, physical operations, and other methods to provide one-on-one training	Control (4w)	Physical therapy and routine work therapy	Cognitive function and activities of daily living	FIM NCSE
		C:18	C:10/8	C:62 ± 4						
(Yeh et al., 2022) Taiwan, China	RCT	E1:20	E1:12/8	E1:53.05 ± 14.53	E1: CBCT_EX E2: EX (60 min/d,3d/ w,12w)	CBCT: BrainHQ software; EX: Progressive resistive stationary bicycle training	CBCT (60 min/d,3d/w,12w)	BrainHQ software	Attention, recognition, color and shape identification, calculation, visual perception, visuospatial processing, memory and executive function	MoCA WMS-III FIM
		E2:18	E2:13/5	E2:57.36 ± 12.17						
		C:18	C:13/5	C:60.17 ± 12.13						
(Wang et al., 2009) China	RCT	E:42	E:23/19	E:55	CT (30min/d,7/w,2m)	Attention training, apraxia training, spatial perception training, and thinking operation ability training	Control (2m)	NA	Cognitive function	BI Fugl- Meyer
		C:42	C:25/17	C:55						
(Gong, 2017) China	RCT	E:33	E:17/16	E:50.88 ± 6.14	CT (20min/d,2d/w,8w)	Attention training, memory training, aphasia training, visual and tactile training and computational power training	Control (8w)	Physical therapy and work therapy	Cognitive function	MoCA MMSE
		C:33	C:18/15	C:55.82 ± 9.21						
(Sun et al., 2022) China	RCT	E:17	E:12/5	E:54.0 (14.0)	CT_EX (CT 40min/d,5d/w,4w) + (EX 40min/d,5d/w,4w)	CT: Calculation, reasoning, working memory, processing speed, executive control, and attention. EX: Exercise training included rehabilitation treadmill training and walking on a flat surface. Motor tasks and cognitive tasks are performed simultaneously	CT (40min/d,5d/w,4w)	Calculation, reasoning, working memory, processing speed, executive control, and attention	Speed of cognitive processing and cognitive function	MoCA MMSE P300
		C:16	C:13/3	C:60.5 (10.6)						
(Yeh et al., 2019) Taiwan, China	RCT	E:15	E:8/7	E: 50.63 (3.99)	CBCT_EX (60 min/d,2-3d/w,12-18w)	CBCT: First underwent aerobic exercise training for 30 minutes, followed by 30 minutes of computer based cognitive training. Computer-based cognitive training with the brainhq program EX: Aerobic exercise training was conducted using a progressive resistance stationary bicycle and 30 minutes of unstructured mental activities that did not train a specific cognitive domain	EX (60 min/d,2-3d/w,12-18w)	Aerobic exercise training was conducted using a progressive resistance stationary bicycle and 30 minutes of unstructured mental activities that did not train a specific cognitive domain	Attention, recognition, color and shape identification, calculation, visual perception, visuospatial processing and executive function	MoCA FIM lawton-LADL WMS-III 6MWT SIS TUG
		C:15	C:13/2	C:60.21 (3.10)						

### 3.2 Quality evaluation of research methods

**FIGURE 2:**
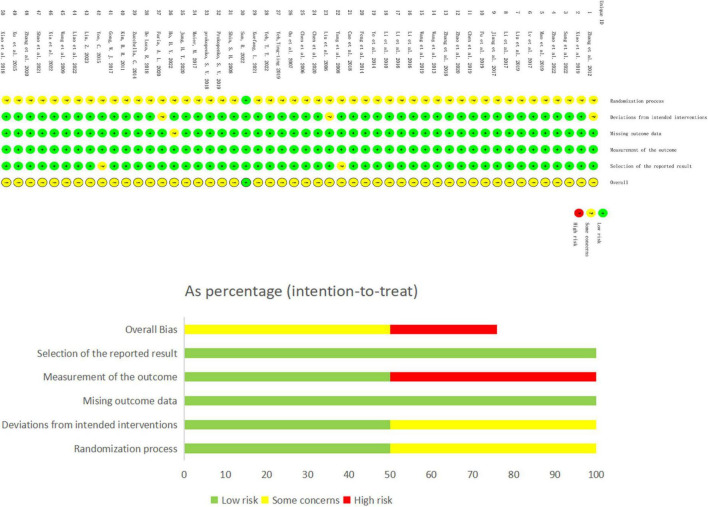
Risk bias assessment plot.

This study included 50 papers. While most studies detailed their randomization process, 13 did not specify the method of random sequence generation. Only one study (Sun et al., 2022), described allocation concealment, leading to the classification of the others as having an ‘unknown risk’ of bias in this regard. Due to the nature of the interventions, blinding of participants was not feasible. A total 13 studies described blinding methods, while the remaining were assessed as having an unknown risk. Concerning baseline data consistency, all studies reported comparable baseline data with no statistical differences, thus receiving a ‘low risk’ rating. In terms of outcome data completeness, most studies accounted for all participants at the time of outcome reporting, aligning with the number initially included and were thus rated as ‘low risk’. However, seven studies reported data loss or dropout rates exceeding 5% of the original sample size, which was assessed as ‘unknown risk’. Three studies were evaluated as ‘unknown risk’ due to the inaccessibility of their original statistical methods. The detailed methodological quality assessment is provided in a table, and the risk bias assessment is illustrated in Figure 2.

### 3.3 Results analysis

**FIGURE 3:**
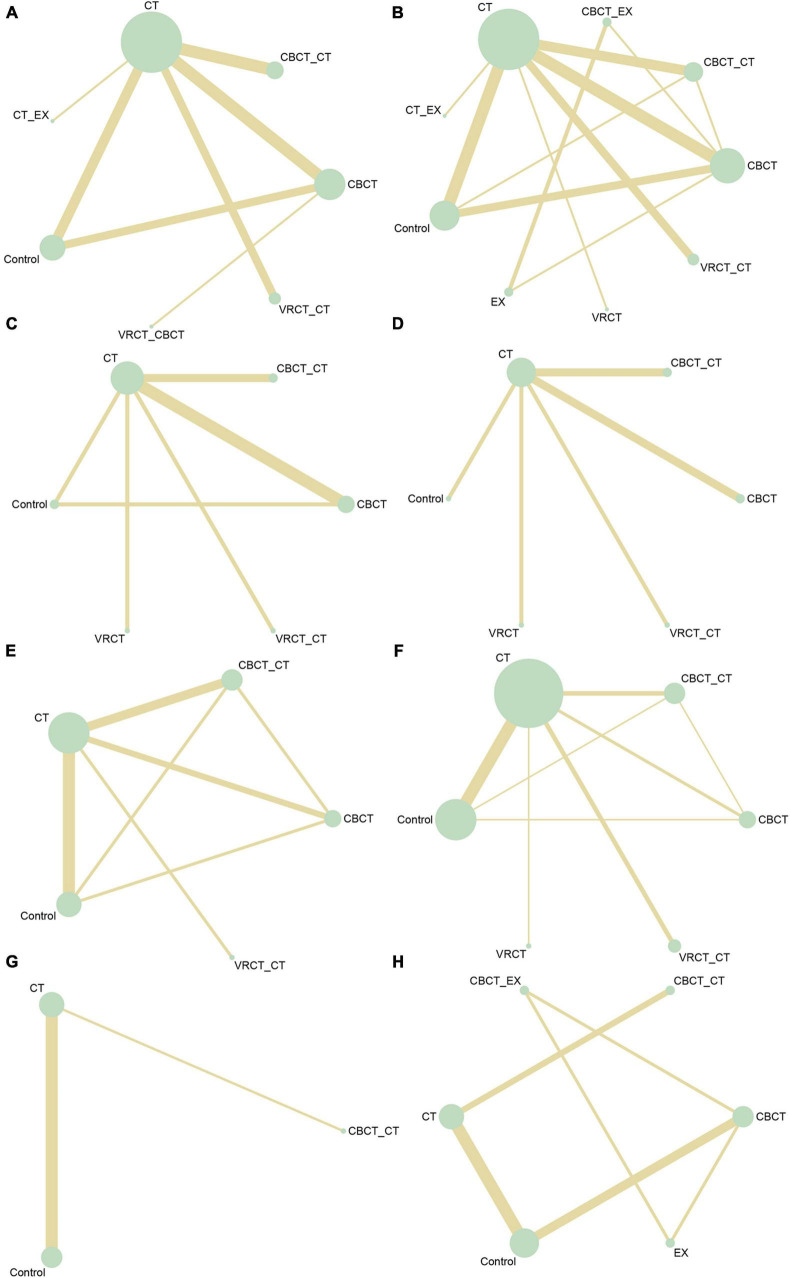
Network intervention comparison network diagram. **(A)** MMSE. **(B)** MoCA. **(C)** Visuo-executive, attention, abstraction, memory, and orientation function in MoCA. **(D)** Naming and language function in MoCA. **(E)** LOTCA. **(F)** BI. **(G)** FMA. **(H)** FIM.

**FIGURE 4:**
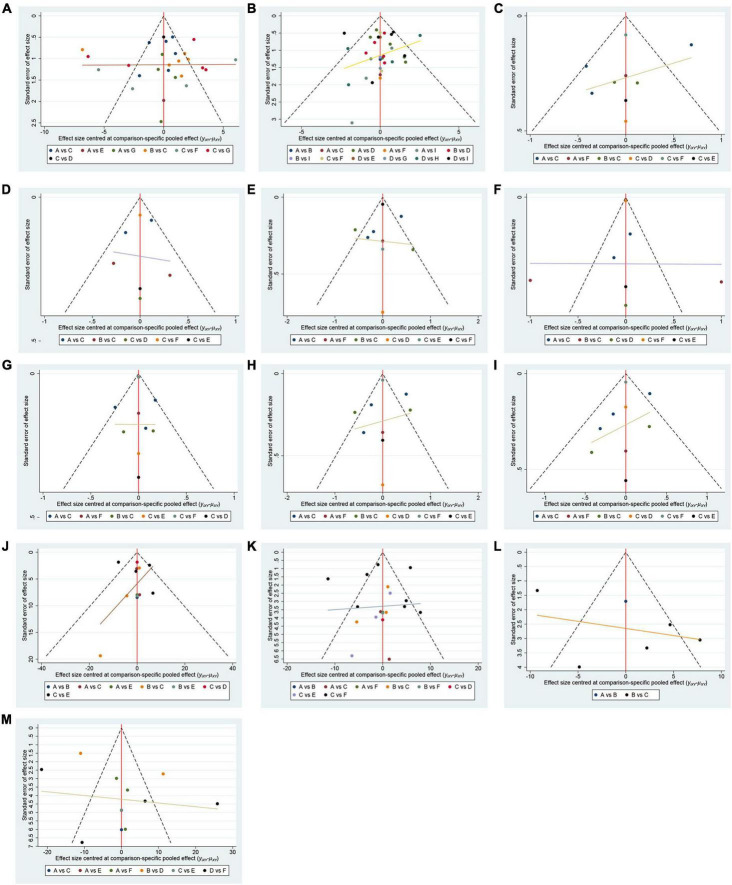
Funnel plot. **(A)** MMSE; A(reference): CBCT; B:CBCT_CT; C:CT; D:CT_EX; E:VRCT_CBCT; F:VRCT_CT; G:Control. **(B)** MoCA; A(reference):CBCT; B:CBCT_CT; C:CBCT_EX; D:CT; E:CT_EX; F:EX; G:VRCT; H:VRCT_CT; I:Control. **(C)** visuo-executive function in MoCA; A (reference):CBCT; B:CBCT_CT; C:CT; D:Control; E:VRCT; F:VRCT_CT. **(D)** naming function in MoCA; A (reference):CBCT; B:CBCT_CT; C:CT; D:Control; E:VRCT; F:VRCT_CT. **(E)** attention function in MoCA; A (reference):CBCT; B:CBCT_CT; C:CT; D:Control; E:VRCT; F:VRCT_CT. **(F)** language function in MoCA; A (reference):CBCT; B:CBCT_CT; C:CT; D:Control; E:VRCT; F:VRCT_CT. **(G)** abstraction function in MoCA; A (reference):CBCT; B:CBCT_CT; C:CT; D:Control; E:VRCT; F:VRCT_CT. **(H)** memory function in MoCA; A (reference):CBCT; B:CBCT_CT; C:CT; D:Control; E:VRCT; F:VRCT_CT. **(I)** orientation function in MoCA;; A (reference):CBCT; B:CBCT_CT; C:CT; D:Control; E:VRCT; F:VRCT_CT. **(J)** LOTCA; A (reference):CBCT; B:CBCT_CT; C:CT; D:VRCT_CT; E:Control. **(K)** BI; A(reference):CBCT; B:CBCT_CT; C:CT; D:VRCT; E:VRCT_CT; F:Control. **(L)** FMA; A (reference):CBCT_CT; B:CT; C:Control. **(M)** FIM; A(reference):CBCT; B:CBCT_CT; C:CBCT_EX; D:CT; E:EX; F:Control.

In total, 50 randomized controlled trials involving 3063 participants were included to assess the impact of various cognitive training methods on cognitive function in patients with PSCI. The network structure diagram, representing various interventions across different outcome indices, is presented in Figure 3. The line thickness in the diagram correlates with the number of pairwise intervention comparisons - the thicker the line, the greater the number of comparisons. Circle size corresponds to the sample size involved in each intervention. The study analyzed both the consistency and inconsistency of data across each outcome index. A difference of less than 5 in the DIC results between the consistency and inconsistency models suggested data consistency for each outcome. Closed loops were observed in the MoCA, MMSE, LOTCA, BI, and FIM scales network diagrams. The node-splitting method was employed for local inconsistency tests on these closed-loop outcomes, yielding P-values all above 0.05, indicating no significant local inconsistency among interventions forming these loops. Publication bias for the included studies was assessed using comparison-correction funnel plots for all outcomes, which demonstrated basic symmetry and a roughly symmetric distribution of studies on both sides of the central line, suggesting a low risk of publication bias (Figure 4).

#### 3.3.1 Overall cognitive function

##### 3.3.1.1 MMSE scale

**FIGURE 5:**
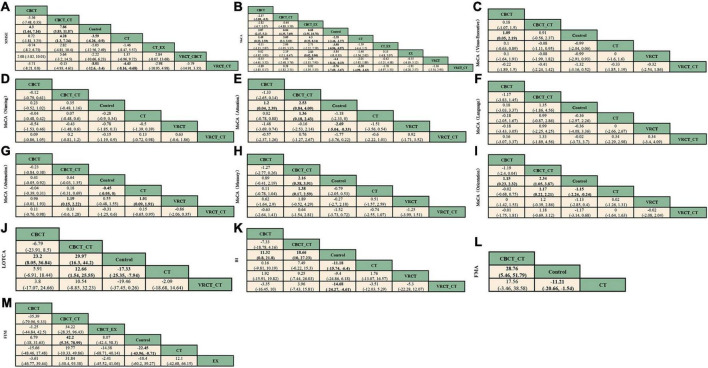
League table. **(A)** MMSE. **(B)** MoCA. **(C)** visuo-executive function in MoCA. **(D)** naming function in MoCA. **(E)** attention function in MoCA. **(F)** language function in MoCA. **(G)** abstraction function in MoCA. **(H)** memory function in MoCA. **(I)** orientation function in MoCA. **(J)** LOTCA. **(K)** BI. **(L)** FMA. **(M)** FIM.

**FIGURE 6:**
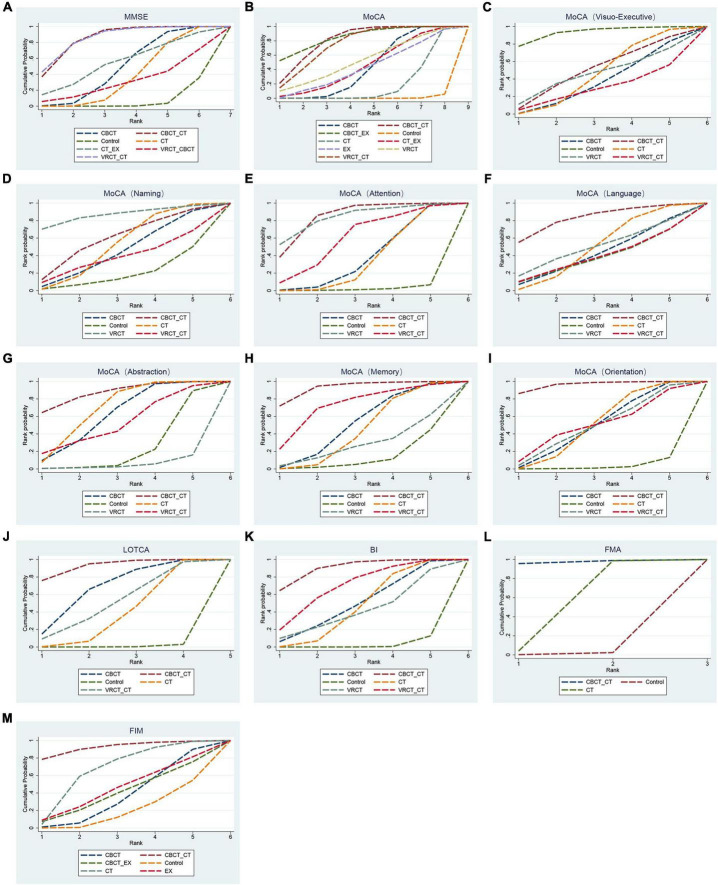
Cumulative sorting chart. **(A)** MMSE. **(B)** MoCA. **(C)** visuo-executive function in MoCA. **(D)** naming function in MoCA. **(E)** attention function in MoCA. **(F)** language function in MoCA. **(G)** abstraction function in MoCA. **(H)** memory function in MoCA. **(I)** orientation function in MoCA. **(J)** LOTCA. **(K)** BI. **(L)** FMA. **(M)** FIM.

The MMSE scale was the focus of 27 studies, encompassing 1497 participants. The network intervention comparison network diagram is depicted in Figure 3A. Four studies assessed the impact of CBCT (Shin et al., 2008; Ye et al., 2014; Prokopenko et al., 2018, 2019), five evaluated traditional cognitive training (Li and Gan, 2010; Zhang et al., 2012; Zucchella et al., 2014; Gong, 2017; Zhao et al., 2020), and 18 directly compared different cognitive training methods (Kim et al., 2011; Wang et al., 2013; Zucchella et al., 2014; Li M. et al., 2016; Cao et al., 2017; Jiang et al., 2017; Lv et al., 2017; Maier et al., 2017; De Luca et al., 2018; Xiao and Liang, 2019; Fu et al., 2020; Jung et al., 2020; Zhang et al., 2020; Zhao et al., 2020; Shao, 2021; Xuefang et al., 2021; Ho et al., 2022; Liao et al., 2022) MMSE score outcomes revealed that the groups receiving CBCT (MD = 4.3, 95% CI:1.44, 7.16, *P* < 0.05), computer-based combined with traditional cognitive training group (MD = 7.86, 95% CI: 3.85, 11.87, *P* < 0.05), VRCT combined with traditional cognitive training (MD = 8.01, 95% CI: 3.4, 12.6, *P* < 0.05), and traditional cognitive training (MD = 3.59, 95% CI: 0.9, 6.26, *P* < 0.05) alone all outperformed the non-cognitive training control group. Additionally, the group with combined computer-based and traditional cognitive training showed greater improvements than the group with traditional cognitive training (MD = 4.28, 95% CI: 1.3, 7.24, *P* < 0.05), as did the group combining VRCT and traditional cognitive training when compared to the traditional training group alone (MD = 4.43, 95% CI: 0.68, 8.16, *P* < 0.05), with these differences being statistically significant. No significant differences were observed in other pairwise comparisons (Figure 5A). Cumulative probability results indicated that the top three interventions for improving MMSE scores were likely VRCT combined with traditional cognitive training (SUCRAs: 85.63%), CBCT combined with traditional cognitive training (SUCRAs: 85.18%), and exercise combined with traditional cognitive training (SUCRAs: 55.12%) (Figure 6A).

##### 3.3.1.2 MoCA scale

In total, 27 randomized controlled trials involving 1680 participants analyzed the MoCA scores of PSCI patients pre- and post-intervention. The intervention comparison network diagram is shown in Figure 3B. Four studies investigated the impact of CBCT (Ye et al., 2014; Prokopenko et al., 2018, 2019; Wang, 2019), five focused on traditional cognitive training (Li and Gan, 2010; Feng et al., 2014; Li X. et al., 2016; Gong, 2017; Zhang et al., 2018), and 18 compared different cognitive training methods (Cao et al., 2017; Xiao, 2018; Chen et al., 2019; Liu et al., 2019, 2022; Mao et al., 2019; Wang, 2019; Xiao and Liang, 2019; Yeh et al., 2019, 2022; Faria et al., 2020; Shao, 2021; Xuefang et al., 2021; Ho et al., 2022; Liao et al., 2022; Shang et al., 2022; Sun et al., 2022; Zhao et al., 2022). Of these, 26 studies used a total score system for assessment, while one study scored only certain aspects of the scale. MOCA score outcomes indicated that groups receiving CBCT combined with traditional cognitive training (MD = 6.04, 95% CI: 4.35, 7.69, *P* < 0.05), CBCT alone (MD = 3.87, 95% CI:2.47, 5.2, *P* < 0.05), exercise combined with CBCT (MD = 6.68, 95% CI:2.55, 10.78, *P* < 0.05), VRCT combined with traditional cognitive training (MD = 5.76, 95% CI:3.67, 7.69, *P* < 0.05), VRCT (MD = 4.44, 95% CI:0.33, 8.44, *P* < 0.05), traditional cognitive training combined with exercise (MD = 3.98, 95% CI:0.97, 6.96, *P* < 0.05), and traditional cognitive training alone were all superior to the control group (MD = 2.38, 95% CI: 1.27, 3.46, *P* < 0.05). Moreover, the group with combined computer-based and traditional cognitive training outperformed the traditional cognitive training group (MD = 3.65, 95% CI: 2.3, 5.02, *P* < 0.05) and CBCT group (MD = 2.17, 95% CI: 0.5, 3.89, *P* < 0.05) alone. The computer-based (MD = 1.49, 95% CI: 0.33, 2.59, *P* < 0.05), exercise combined with computer-based (MD = 4.3, 95% CI: 0.25, 8.34, *P* < 0.05), and VRCT combined with traditional cognitive training groups (MD = 3.38, 95% CI: 1.63, 4.99, *P* < 0.05) all showed superior results compared to the traditional cognitive training group, and the group combining exercise with CBCT (MD = 2.83, 95% CI:0.62, 5.06, *P* < 0.05) also outperformed the exercise group alone, with these differences being statistically significant. No significant differences were found in other pairwise comparisons (Figure 5B). The ranking of interventions is provided in Figure 4. Cumulative probability results identified the top three interventions as exercise combined with traditional cognitive training (SUCRAs: 85.40%), CBCT combined with traditional cognitive training (SUCRAs: 81.54%), and VRCT combined with traditional cognitive training (SUCRAs: 76.30%) (Figure 6B).

Nine studies reported therapeutic effects across seven distinct cognitive domains (Xiao, 2018; Zhang et al., 2018; Liu et al., 2019; Xiao and Liang, 2019; Faria et al., 2020; Shao, 2021; Shang et al., 2022; Xia et al., 2022; Zhao et al., 2022). In visual-spatial and executive function, the intervention comparison network diagram is shown in Figure 3C. The CBCT group (MD = 1.09, 95% CI:0.05, 2.19, *P* < 0.05) outperformed the conventional control group, with a statistically significant difference (Figure 5C). The cumulative sorting chart is shown in Figure 6C. Regarding naming and language function, the intervention comparison network diagram is shown in Figure 3D. No intervention demonstrated a significant impact on the recovery of these functions in PSCI patients (Figures 5D, E). The cumulative sorting chart is shown in Figures 6D, E.

For attention abilities, the intervention comparison network diagram is shown in Figure 3C. The groups receiving CBCT combined with traditional cognitive training (MD = 2.53, 95% CI:0.84, 4.09, *P* < 0.05), CBCT alone (MD = 1.2, 95% CI:0.04, 2.39, *P* < 0.05), and VRCT (MD = 2.69, 95% CI:0.33, 5.04, *P* < 0.05) all showed superior results compared to the conventional control group, with these differences being statistically significant (Figure 5F). Cumulative probability results suggest that CBCT combined with traditional cognitive training (SUCRAs: 84.17%) and VRCT (SUCRAs: 83.46%) might be the most effective interventions for improving attention abilities (Figure 6F).

In terms of abstract function, the intervention comparison network diagram is shown in Figure 3C. The traditional cognitive training group (MD = 0.45, 95% CI:0,0.95, *P* < 0.05) exceeded the conventional control group’s performance, and both the CBCT combined with traditional cognitive training (MD = 1.19, 95% CI:0.15, 2.22, *P* < 0.05) and the cognitive training groups (MD = 1.01, 95% CI:0.09, 1.91, *P* < 0.05) outperformed the VRCT group (Figure 5G). The cumulative sorting chart is shown in Figure 6G.

For delayed recall, the intervention comparison network diagram is shown in Figure 3C. The group combining computer-based and traditional cognitive training showed better results than both the conventional control (MD = 2.16, 95% CI:0.38, 3.91, *P* < 0.05) and traditional cognitive training groups (MD = 1.38, 95% CI:0.17, 2.59, *P* < 0.05) (Figure 5H). The cumulative sorting chart is shown in Figure 6H. In orientation function, the intervention comparison network diagram is shown in Figure 3C. The groups receiving CBCT combined with traditional cognitive training (MD = 2.34, 95% CI:1.05, 3.87, *P* < 0.05), CBCT alone (MD = 1.15, 95% CI:0.23, 2.32, *P* < 0.05), and traditional cognitive training (MD = 1.15, 95% CI:0.24, 2.24, *P* < 0.05) all surpassed the conventional control group. Additionally, the combination of computer-based and traditional cognitive training was more effective than traditional cognitive training alone (MD = 1.17, 95% CI:0.22, 2.21, *P* < 0.05), with a statistically significant difference (Figure 5I). The cumulative sorting chart is shown in Figure 6I.

In conclusion, based on cumulative probability outcomes, CBCT combined with traditional cognitive training is the leading intervention for enhancing attention, abstract function, memory, and orientation, as measured by the MoCA Scale.

##### 3.3.1.3 LOTCA scale

Eight studies, encompassing 476 participants, were included for analysis. The intervention comparison network diagram is displayed in Figure 3E. One study assessed the impact of CBCT (Wang, 2019), three studies evaluated traditional cognitive training (Chen et al., 2006, 2020; Wang et al., 2009), and four studies directly compared various cognitive training methods (Wang et al., 2013; Li M. et al., 2016; Wang, 2019; Xuefang et al., 2021). LOTCA score outcomes indicated that the groups receiving CBCT combined with traditional cognitive training (MD = 29.97, 95% CI:16.3, 44.2, *P* < 0.05), CBCT alone (MD = 23.2, 95% CI:8.05, 36.84, *P* < 0.05), and traditional cognitive training (MD = 17.33, 95% CI:7.94, 25.35, *P* < 0.05) all surpassed the control group, with these differences being statistically significant (Figure 5J). Cumulative probability results identified CBCT combined with traditional cognitive training (SUCRAs: 92.64%), CBCT (SUCRAs: 67.16%), and VRCT combined with traditional cognitive training (SUCRAs: 51.00%) as the top three interventions (Figure 6J). The combination of computer-based and traditional cognitive training demonstrated the most substantial impact on LOTCA scale scores.

##### 3.3.1.4 Neurocognitive and neuropsychological assessment

Neurocognitive and neuropsychological testing serve as crucial instruments for evaluating cognitive domains, encompassing a broad spectrum of cognitive functions such as, but not limited to, perception and visuospatial function, motor control, attention, memory, executive function, language, and intellectual quotient (Zucchella et al., 2018). In studies that employed Digit span (forward and backward) tests to detect patients’ short-term auditory memory and working memory capacity, six out of eight studies revealed that Digit span tests demonstrated certain clinical amelioration subsequent to the administration of these cognitive training (Zucchella et al., 2014; Yoo et al., 2015; Xiao, 2018; Jung et al., 2020; Ho et al., 2022; Liu et al., 2022). Moreover, Digit span backward test often showed statistical significance more often than Digit span forward test. Two studies demonstrated no significant enhancement (Kim et al., 2011; Faria et al., 2020). In assessments employing the Trail-making test (parts A and B) to investigate attention processes in individuals with PSCI, outcomes from five of the eight studies indicated that there was also a clinical improvement in attention following these cognitive training interventions, particularly in the Trail-making test parts A outcome (Zucchella et al., 2014; Maier et al., 2017; Xiao, 2018; Faria et al., 2020; Liu et al., 2022). Three studies did not demonstrate significant improvement following these cognitive interventions (Kim et al., 2011; Yoo et al., 2015; De Luca et al., 2018). Additionally, one study discovered that in the improvement phase Digit span tests, the combination of VRCT with traditional cognitive training demonstrated superior clinical efficacy compared to the traditional cognitive training group alone, and this difference was statistically significant (Jung et al., 2020). In another study, the traditional cognitive training group exhibited advantages over traditional rehabilitation therapy in both the Digit span tests and Trail-making test assessments (Zucchella et al., 2014).

#### 3.3.2 Function of daily living

Fifteen studies, involving 937 patients, reported on the effects of various interventions on the BI scores of PSCI patients. The intervention comparison network diagram is illustrated in Figure 3F. One study investigated the impact of CBCT (Wang, 2019), seven studies focused on traditional cognitive training (Liu et al., 2006; Ou et al., 2007; Wang et al., 2009; Zhang et al., 2012, 2018; Feng et al., 2014; Zhao et al., 2020), and eight studies directly compared different cognitive training methods (Maier et al., 2017; Xiao, 2018; Chen et al., 2019; Liu et al., 2019, 2022; Mao et al., 2019; Wang, 2019; Xiao and Liang, 2019). BI index results showed that the groups receiving CBCT combined with traditional cognitive training (MD = 18.66, 95% CI:10, 27.23, *P* < 0.05), VRCT combined with traditional cognitive training (MD = 14.68, 95% CI:4.61, 24.27, *P* < 0.05), CBCT alone (MD = 11.32, 95% CI:0.8, 21.8, *P* < 0.05), and traditional cognitive training (MD = 11.18, 95% CI:6.4, 15.74, *P* < 0.05) all achieved higher scores than the control group, with these differences being statistically significant. No significant differences were found in other pairwise comparisons (Figure 5K). Cumulative probability results revealed that CBCT combined with traditional cognitive training (SUCRAs: 90.19%), VRCT combined with traditional cognitive training (SUCRAs: 69.36%), and CBCT (SUCRAs: 49.59%) were the top-ranked interventions (Figure 6K), suggesting their greater effectiveness in improving the daily living functions of PSCI patients.

#### 3.3.3 Motor function

Six studies, using FMA score to assess the effects of motor function, totaling 426 participants, were included for analysis. The intervention comparison network diagram is presented in Figure 3G. Five studies evaluated the impact of traditional cognitive training (Chen et al., 2006, 2020; Ou et al., 2007; Wang et al., 2009; Li and Gan, 2010), and one study directly compared CBCT combined with traditional cognitive training to traditional cognitive training alone (Lv et al., 2017). FMA score results indicated that both the group receiving CBCT combined with traditional cognitive training (MD = 28.76, 95% CI:5.46, 51.79, *P* < 0.05) and the traditional cognitive training group (MD = 11.21, 95% CI:1.54, 20.66, *P* < 0.05) outperformed the control group, with statistically significant differences (Figure 5L). The combined computer-based and traditional cognitive training approach ranked as the most effective intervention (Figure 6L), demonstrating a pronounced effect on improving motor function in patients with PSCI.

#### 3.3.4 Functional independence

Ten studies, using FIM score to assess the effects of functional independence, encompassing 560 patients, were included in this segment. The intervention comparison network diagram is depicted in Figure 3H. Three studies assessed the impact of CBCT (Shin et al., 2008; Ye et al., 2014; Yoo et al., 2015), four studies focused on traditional cognitive training (Chen et al., 2006, 2020; Liu et al., 2006; Yang and Liu, 2008; Zucchella et al., 2014), and three studies directly compared different cognitive training methods (Lv et al., 2017; Liu et al., 2019; Yeh et al., 2022). The results showed that both the group receiving CBCT combined with traditional cognitive training (MD = 42.2, 95% CI:5.25, 78.99, *P* < 0.05) and the traditional cognitive training group (MD = 22.45, 95% CI:0.71, 43.96, *P* < 0.05) significantly outperformed the conventional control group in enhancing functional independence. There were no notable differences in other pairwise interventions (Figure 5M), with the combination of computer-based and traditional cognitive training (SUCRAs: 92.16%) proving to be the most effective (Figure 6M).

## 4 Discussion

### 4.1 Explanation of the results and comparison with previous studies

To our knowledge, this study represents the first network meta-analysis comparing various cognitive training treatments for PSCI patients. It encompassed 50 randomized controlled trials involving 3017 subjects, analyzing nine cognitive interventions. Our study found that cognitive training can positively affect cognitive function, daily living function, functional independence and motor function in PSCI patients. The combination of CBCT and traditional cognitive training demonstrated the most significant impact on overall cognitive function improvement as assessed by the MMSE and LOTCA scales, ranking second only to the exercise combined CBCT group as assessed by the MoCA scale. The combined CBCT and traditional cognitive training group showed significant improvements in attention, abstract ability, memory, and orientation compared to other training groups. Furthermore, it ranked highest in terms of impact on daily living function as measured by the BI index, FMA-based daily living function, and FMI-based functional independence. This study primarily revolved around these simplified cognitive screening instruments and life function scales, which facilitate rapid and straightforward assessments of patients’ cognitive functions. Although the obtained results might be approximate, such screening tests are adequate when scores are low and the patient’s medical history strongly indicates dementia, along with staging and monitoring of cognitive impairment. Of course, within the literature we examined, several studies utilizing a variety of neurocognitive and neuropsychological measures have been conducted to uncover the potential benefits of cognitive training on various cognitive dimensions in patients suffering from PSCI. However, due to the heterogeneity of neuropsychological tests employed across studies, the limited recurrence of the same test, and the subtle variances in the focus of interventions and anticipated outcomes, we did not extract the results of specific tests individually and perform meta-analyses. Instead, we adopted a more macro and inclusive methodological strategy, aiming to capture the overall trend of cognitive training enhancing cognitive function in PSCI patients as a whole. By reviewing these articles, we discovered that the majority of studies employed the Digit Span (forward and backward) tests to assess patients’ short-term auditory memory and working memory capacity, as well as the Trail-Making Test (parts A and B) to evaluate the attention process of PSCI patients. Following the administration of these cognitive interventions, there was an improvement in clinical efficacy, but the statistically significant difference between groups was rare. Additionally, our study revealed that CBCT in conjunction with traditional cognitive training yielded a more significant impact on enhancing attention and memory compared to traditional cognitive training or rehabilitation alone. This finding contrasts slightly with the results of De Luca et al. (2018) and Xiao (2018) studies, which did not demonstrate a distinct advantage for the CBCT combined with traditional cognitive training group. This might be associated with the scale employed. The assessment of memory and attention using the MoCA scale is frequently simplistic and approximate, which can easily exaggerate or overlook the cognitive function scores of patients, leading to conspicuous data disparities. Conversely, neuropsychological tests are typically more meticulous and intricate, enabling the assignment of more precise scores and minimizing potential errors.

The inaugural ‘Adult Stroke Rehabilitation Guideline’ issued jointly by the American Heart Association (AHA) and the American Stroke Association (ASA) in 2016 recommended cognitive function rehabilitation training for post-stroke patients at a Level IA (Winstein et al., 2016). Jeffrey M. Rogers et al.’s systematic review highlighted that cognitive training effectively enhances cognitive function in PSCI patients. Our study’s findings align with these perspectives. In the 50 randomized controlled trials employing various cognitive training interventions for PSCI patients, we observed that both traditional cognitive training and CBCT combined with traditional cognitive training improved all study outcome indicators. These interventions notably enhanced overall cognitive function and daily living abilities. Furthermore, compared to traditional cognitive training alone, combined interventions often yielded greater cognitive benefits and effectiveness. Specifically, CBCT combined with traditional cognitive training emerged as the most effective in enhancing attention, abstract function, memory, orientation, daily living function, motor function, and functional independence. Our analysis revealed consistent results between the MMSE and MoCA scales. The top three interventions were identified as a combination of cognitive training and CBCT with traditional cognitive training, VRCT combined with traditional cognitive training, and exercise combined with CBCT. It suggests that these three combined interventions have advantages in improving the overall cognitive function of PSCI patients.

CBCT offers personalized and adaptive content tailored to the patient’s specific cognitive impairments, adjusting in real-time to the feedback received during training sessions. This adaptability ensures that tasks are neither too simple nor too complex, thereby enhancing patient engagement and concentration in the training process (Chuang et al., 2023; Maggio et al., 2023). The study observed that older adults demonstrate greater enthusiasm and compliance for CBCT compared to general cognitive training. Effective cognitive training requires participants to fully commit to the regimen to achieve an adequate ‘training dose’ and thereby reap the benefits (Turunen et al., 2019), which supports the finding that CBCT tends to result in better outcomes. The research indicates that computer games used in this training not only enhance visual-spatial abilities but also improve attention. This is achieved by training the spatial and temporal resolution of attention in these games, thus enhancing attention control and task focus (Bavelier and Green, 2019). Research by Richard E. Mayer suggests that games focusing on a single cognitive skill can enhance cognitive functions such as memory, attention, and spatial cognition. This enhancement requires participants to repeatedly practice the skill in varied environments and with progressively increasing challenges (Mayer, 2019). Cognitive training operates on repeated practice in specific cognitive domains, using tasks involving particular skills to achieve training objectives. Cognitive training is founded on the repetition of specific cognitive domain training, and the intended outcome is realized through the execution of tasks that necessitate specific skills. This renders CBCT, a training approach that incorporates computer games, more effective in enhancing the attention, memory, and visuospatial abilities of PSCI patients. This is in line with the fact that CBCT, alone or in conjunction with traditional cognitive training, can enhance visuospatial and executive function, attention, memory, and orientation. However, despite their adaptability, these technological tools cannot entirely replace traditional cognitive training, especially for more severe and subacute cases, where the role of neuropsychiatrists and speech therapists is crucial. In fact, combining computer-based with traditional cognitive training often results in optimal outcomes (Maggio et al., 2023). A meta-analysis of 32 studies involving 1837 subjects corroborates this study’s findings, revealing that combining computer-based and traditional cognitive training significantly enhances overall cognitive function and daily life function more than conventional cognitive training alone (Nie et al., 2022). In summary, CBCT synergistic intervention demonstrates effectiveness in enhancing cognitive function and mitigating neurological deficits in patients with PSCI. The underlying mechanism is potentially associated with enhanced cerebral blood flow, increased serum BDNF levels, suppressed VILIP-1 and NSE expression, and the repair of damaged neurons. These factors contribute to improving patient prognosis and accelerating the recovery process (Xuefang et al., 2021).

VRCT, akin to CBCT, offers high adaptability by providing personalized training tailored to the participant’s cognitive and physical conditions. This training method features enhanced ecological validity, creating a three-dimensional, digital environment where subjects engage in cognitive tasks within virtual recreations of familiar daily activities (Moreno et al., 2019). Ana Lúcia Faria et al.’s study highlighted that VRCT yields greater cognitive benefits than traditional methods (Faria et al., 2020), aligning with this study’s findings. The effectiveness of VRCT may stem from its immersive experience. By engaging participants in situational daily life scenarios, it offers multisensory, natural, and realistic stimulation. This immersion significantly heightens patients’ action awareness, self-identity, and self-cognition, fostering high interest and enjoyment in VR usage (Choi et al., 2019), thereby comprehensively exercising their abilities and enhancing task success through timely feedback (Faria et al., 2020). Additionally, the gaming aspect of VRCT can also improve cognitive functions. However, it’s noteworthy that participants tend to learn better on desktop computer screens compared to VR games (Mayer, 2019), potentially explaining why CBCT is more effective than its VR counterpart. To elucidate the advantages of VRCT, researchers have proposed various mechanisms. Carrieri et al. suggested that VR can stimulate the reactivation and enhancement of diverse cortical functions, particularly the dorsolateral/ventrolateral prefrontal cortex, optimize sensory cortex efficiency, and boost cognitive function (Carrieri et al., 2016). Flannery et al. noted that VRCT training activates brain metabolism, increases cerebral blood flow, and enhances neurotransmitter release (Flannery, 2002).

Sports combined with cognitive training encompasses two modalities: sequential exercise and cognitive training, and dual-task cognitive training, which concurrently emphasizes physical and cognitive training. This approach is more apt for enhancing the comprehensive skills required in daily life. Prior research indicates that such training is more advantageous in improving overall cognition, working memory, and executive function in elderly individuals with cognitive impairment than conventional treatments (Guo et al., 2020). I-Ching Chuang et al. discovered that exercise combined with cognitive training is more beneficial in enhancing overall cognitive function, particularly in visual-spatial working memory and language memory, compared to either physical or cognitive training alone. However, this approach does not demonstrate significant advantages in daily living abilities (Chuang et al., 2023), aligning with this study’s findings. It is posited that both simultaneous and sequential exercise and cognitive training may impact different cognitive aspects (McEwen et al., 2018). While there is ongoing debate regarding the superiority of each training mode, it is established that exercise combined with cognitive training constitutes an effective intervention. This efficacy could be attributed to exercise’s role in elevating brain neurotrophic factors in the peripheral blood (Marinus et al., 2019), crucial for the survival, maintenance, and regeneration of specific brain neuron populations (Allen et al., 2013). There is evidence indicating that physical exercise enhances the volume of the prefrontal cortex and the prehippocampus, fosters nerve and angiogenesis, and plays a crucial role in reducing cardiovascular risk factors. Furthermore, it has been discovered that the combination of exercise and cognitive training often yields more substantial benefits than either exercise or cognitive training alone. The neurological and cognitive benefits derived from exercise may be associated with increased environmental exposure during cognitive challenge (Karssemeijer et al., 2017). While environmental enrichment is considered to enhance language development in young children, improve cognitive abilities, and reduce the risk of dementia. Increased physical engagement in the form of exercise can enhance cognitive performance, mitigate memory impairment, and decrease the likelihood of Alzheimer’s disease in older adults (Figuracion and Lewis, 2021). In 2016, the inaugural “Adult Stroke Rehabilitation Guidelines” jointly published by the AHA and the ASA also issued Grade-A recommendations for cognitive activities within environmentally enriched settings (Winstein et al., 2016). However, only three related studies were included. Additionally, only the cognitive outcome of the MoCA scale demonstrates that exercise in conjunction with CBCT yields a more favorable effect than CBCT combined with traditional cognitive training. Further high-quality randomized controlled studies are required to ascertain the superiority and inferiority of exercise in combination with CBCT and CBCT in conjunction with traditional cognitive training in enhancing cognitive function and daily living abilities.

Regarding the improvement of motor function, interventions involving CBCT combined with traditional cognitive training are significant. The identical outcomes were obtained in the research, where the functional independence rating served as the outcome index. It should be highlighted that only CBCT in combination with traditional cognitive training, traditional cognitive training, and the conventional control group were incorporated in the study evaluating motor function. Both interventions demonstrated significant improvements compared to the conventional control group. The enhancement of motor function induced by CBCT and traditional cognitive training might be associated with the following reasons. One is the unique training mode of CBCT. CBCT engages participants in using specialized buttons and joysticks to exercise the movements of the wrist, thumb, and index finger of the impaired hand. This approach allows participants to enhance hand flexibility while engaging in cognitive tasks, thereby improving limb coordination in post-stroke patients (Otfinowski et al., 2006), which may account for the reason why the combination of CBCT with traditional cognitive training enhance motor function more effectively than traditional cognitive training alone. It was widely recognized that there is a connection between movement disorders and cognitive decline, although the underlying mechanism remains incompletely understood (Kobayashi-Cuya et al., 2018). In a prospective cohort study conducted by Zhangyu Wang et al., MRI was used to evaluate brain structural volume (including total brain volume, total white matter, total gray matter, hippocampus volume, and white matter high signal) and statistically analyzed in conjunction with motor function and cognitive function data. Ultimately, it was demonstrated that motor impairment is associated with declines in cognitive functions such as overall cognition, episodic memory, semantic memory, working memory, visuospatial ability and perceptual speed (Wang et al., 2023). Paul Rinne et al. identified a consistent and strong correlation between attention control and motor performance. They posit that poor attention control following stroke is one of the causes of limb paralysis after stroke, furthermore they suggest that attention control itself may be a therapeutic target for improving motor disorders post-stroke (Rinne et al., 2018). Additionally, previous research has shown that most patients with left hemisphere damage and a minority with right hemisphere damage exhibit impaired ipsilateral hand dexterity within a month post-infarction involving the parietal or posterior frontal lobes. This impairment may be linked to post-stroke cognitive impairment affecting action perception and control (Sunderland et al., 1999). Paul Rinne and colleagues interpreted this to mean that motor function impairment on the same side of the stroke focus was selectively associated with the lesion load on the attention network, but not with the anterior corticospinal tract (Rinne et al., 2018). In view of the relationship between motor function and cognitive function, it is possible that with the improvement of cognitive function, the motor function of PSCI patients will also be restored. Although the link/correlation is not clear and not necessarily causative.

In the assessment of cognitive domains using the MOCA scale, it is indicated that aside from language and naming functions, various cognitive training methods positively influence the improvement of visual-spatial and executive functions, attention, abstraction, delayed recall, and orientation. CBCT combined with traditional cognitive training is most effective in enhancing attention, abstract function, memory, and orientation. These results fit with the advantages brought by the unique cognitive training mode of CBCT. However, our findings suggest that various cognitive training programs did not show any advantage in language or naming. In contrast, computer-assisted cognitive training demonstrates superior benefits in improving visual-spatial and executive functions. This slightly diverges from Anastasia Nousia et al.’s study, which found that CBCT enhances language fluency, naming, and delayed memory (Nousia et al., 2021). Our study exclusively analyzed the various cognitive areas of the MoCA scale, suggesting the necessity for multiple outcome indicators within each cognitive domain, as improvements in a few indicators for the same function are less convincing than enhancements across multiple indicators within one function.

Our results align with most studies under similar outcome indicators, yet they diverge from some. A study affirmed the efficacy of CBCT and cognitive training in enhancing cognition in PSCI patients but did not demonstrate the superiority of CBCT in cognitive rehabilitation of post-stroke patients (Mingming et al., 2022). Compared to our study, their sample was smaller, encompassing only four studies from 2013-2021 with 96 participants, which may introduce bias. A network meta-analysis involving 1047 PSCI patients from 21 RCTs indicated that computer-based and VR-based cognitive training were superior to conventional training in MOCA, with the computer-based group showing the best therapeutic effect. However, this did not reach statistical significance under the MMSE index (Xiao et al., 2022). This study posits that, aside from combined interventions, CBCT surpasses conventional training in MoCA, possibly due to the limited number of studies directly comparing VRCT with traditional training included in this research.

### 4.2 Advantages and limitations of this study

This research entailed an extensive review of studies assessing the efficacy of various cognitive training methods in improving cognitive function in patients with post-stroke cognitive impairment (PSCI). The outcome measures included assessments of cognitive function, daily living, motor function, and functional independence. Given the large sample size and the narrow confidence intervals in this network meta-analysis, we consider the results to be reliable. A key strength of this study is its analysis of diverse cognitive training modalities and detailed examination of different cognitive domains, aspects not covered in previous meta-analyses of cognitive training for PSCI. However, there are several limitations. Firstly, there was no analysis of the heterogeneity of baseline characteristics. The included studies varied in intervention duration and frequency, total intervention time, measurement timings, and the computer platforms used in computer-based and VRCT, contributing to significant heterogeneity. Secondly, only three studies on sports cognitive training were included, with a relatively small sample size, raising concerns about the applicability and accuracy of the results. Thirdly, although all included randomized controlled trials provided conventional drug therapy and rehabilitation training in addition to cognitive training interventions, and studies significantly impacted by drug effects on cognitive function were excluded, the specific roles of these treatments remain ambiguous. This lack of clarity is due to insufficient details about the types and dosages of drugs used post-stroke. Fourthly, the influence of VRCT, which often involves whole-body engagement, is challenging to separate into effects attributable to cognitive training versus physical exercise. Fifthly, the included studies did not adequately describe allocation concealment, and since blinding participants to the interventions was unfeasible, only a few studies blinded the raters. Therefore, the study may contain some subjective biases. Finally, since 66% of the original studies included in this study were in Chinese, the extrapolation of the conclusions of this study has certain limitations.

## 5 Conclusion

Findings from this study demonstrate that supplementing traditional cognitive training with additional training modalities yields superior outcomes in enhancing the cognitive function, daily living skills, functional independence, and motor capabilities in patients with PSCI compared to exclusive cognitive training. Specifically, the influence of an intervention combining CBCT outperforms others in enhancing cognition, daily living function, motor skills, and functional independence. However, an increased number of high-quality, multi-center, large-sample randomized controlled trials are needed in future studies to validate the efficacy of cognitive training. These trials should provide evidence-based data for healthcare professionals to conduct professional assessments based on patients’ specific conditions, and selectively implement these potential non-pharmaceutical interventions in the daily care and rehabilitation of patients with PSCI.

## Data availability statement

The original contributions presented in the study are included in the article/Supplementary material, further inquiries can be directed to the corresponding author.

## Author contributions

LZ: Writing−original draft. XH: Writing−original draft. JW: Writing−review and editing. FW: Writing−review and editing. JL: Writing−review and editing. NL: Writing−review and editing.
