# Supplementary material for: The influence of eight cognitive training regimes upon cognitive screening tool performance in post-stroke survivors: a network meta-analysis
Source: Front Aging Neurosci. 2024 Jul 19;16:1374546. doi: 10.3389/fnagi.2024.1374546 (PMC11294113; doi:10.3389/fnagi.2024.1374546)
Supplement: Supplementary file 1 [file Table_1.DOCX]

# Appendix 1. Literature search strategy

**1.Pubmed**

| Search number | Query |
| --- | --- |
| #1 | "stroke"[MeSH Terms] |
| #2 | "apoplex*"[Title/Abstract] OR "vascular accident*"[Title/Abstract] OR "stroke*"[Title/Abstract] OR "brain attack"[Title/Abstract] OR "brain insult*"[Title/Abstract] OR "cerebral*"[Title/Abstract] OR "cerebrovascular"[Title/Abstract] OR "ischemic seizure"[Title/Abstract] |
| #3 | "cognitive dysfunction"[MeSH Terms] |
| #4 | "Cognitive Decline"[Title/Abstract] OR "amnestic"[Title/Abstract] OR "cognition disorder*"[Title/Abstract] OR "cognitive defect*"[Title/Abstract] OR "cognitive deficit"[Title/Abstract] OR "cognitive disability"[Title/Abstract] OR "cognitive disorder*"[Title/Abstract] OR "cognitive dysfunction*"[Title/Abstract] OR "cognitive impairment*"[Title/Abstract] OR "delirium"[Title/Abstract] OR "dementia"[Title/Abstract] OR "mental deterioration*"[Title/Abstract] OR "overinclusion"[Title/Abstract] OR "response interference"[Title/Abstract] |
| #5 | "cognitive training"[MeSH Terms] |
| #6 | "brain training"[Title/Abstract] OR "cognitive rehabilitation"[Title/Abstract] OR "cognitive training"[Title/Abstract] OR "Memory Training"[Title/Abstract] |
| #7 | ("apoplex*"[Title/Abstract] OR "vascular accident*"[Title/Abstract] OR "stroke*"[Title/Abstract] OR "brain attack"[Title/Abstract] OR "brain insult*"[Title/Abstract] OR "cerebral*"[Title/Abstract] OR "cerebrovascular"[Title/Abstract] OR "ischemic seizure"[Title/Abstract] OR "stroke"[MeSH Terms]) AND ("Cognitive Decline"[Title/Abstract] OR "amnestic"[Title/Abstract] OR "cognition disorder*"[Title/Abstract] OR "cognitive defect*"[Title/Abstract] OR "cognitive deficit"[Title/Abstract] OR "cognitive disability"[Title/Abstract] OR "cognitive disorder*"[Title/Abstract] OR "cognitive dysfunction*"[Title/Abstract] OR "cognitive impairment*"[Title/Abstract] OR "delirium"[Title/Abstract] OR "dementia"[Title/Abstract] OR "mental deterioration*"[Title/Abstract] OR "overinclusion"[Title/Abstract] OR "response interference"[Title/Abstract] OR "cognitive dysfunction"[MeSH Terms]) AND ("brain training"[Title/Abstract] OR "cognitive rehabilitation"[Title/Abstract] OR "cognitive training"[Title/Abstract] OR "Memory Training"[Title/Abstract] OR "cognitive training"[MeSH Terms]) |

**2.Cochrane**

| Search number | Query |
| --- | --- |
| #1 | MeSH descriptor: [Stroke] explode all trees |
| #2 | (‘apoplex*’ OR ‘Vascular Accident*’ OR ‘Stroke*’ OR ‘brain accident’ OR ‘brain attack’ OR ‘brain blood flow disturbance’ OR ‘brain insult*’ OR ‘cerebral*’ OR ‘cerebrovascular’ OR ‘ischemic seizure’ OR ‘ischaemic seizure’ ):ti,ab,kw |
| #3 | MeSH descriptor: [Cognitive Dysfunction] explode all trees |
| #4 | (‘Cognitive Decline’ OR ‘amnestic’ OR ‘cognition disorder*’ OR ‘cognitive defect*’ OR ‘cognitive deficit’ OR ‘cognitive disability’ OR ‘Cognitive Disorder*’ OR ‘Cognitive Dysfunction*’ OR ‘Cognitive Impairment*’ OR ‘delirium’ OR ‘dementia’ OR ‘Mental Deterioration*’ OR ‘overinclusion’ OR ‘response interference’ ):ti,ab,kw |
| #5 | MeSH descriptor: [Cognitive Training] explode all trees |
| #6 | (‘brain training’ OR ‘cognitive rehabilitation’ OR ‘cognitive training’ OR ‘Memory Training’ ):ti,ab,kw |
| #7 | (#1 OR #2) AND (#3 OR #4) AND (#5 OR #6) |

**3.Embase**

| Search number | Query |
| --- | --- |
| #1 | 'cerebrovascular accident'/exp |
| #2 | 'apoplex*':ti,ab,kw OR 'vascular accident*':ti,ab,kw OR 'stroke*':ti,ab,kw OR 'brain accident':ti,ab,kw OR 'brain attack':ti,ab,kw OR 'brain blood flow disturbance':ti,ab,kw OR 'brain insult*':ti,ab,kw OR 'cerebral*':ti,ab,kw OR 'cerebrovascular':ti,ab,kw OR 'ischemic seizure':ti,ab,kw OR 'ischaemic seizure':ti,ab,kw |
| #3 | 'cognitive defect'/exp |
| #4 | 'cognitive decline':ti,ab,kw OR 'amnestic':ti,ab,kw OR 'cognition disorder*':ti,ab,kw OR 'cognitive defect*':ti,ab,kw OR 'cognitive deficit':ti,ab,kw OR 'cognitive disability':ti,ab,kw OR 'cognitive disorder*':ti,ab,kw OR 'cognitive dysfunction*':ti,ab,kw OR 'cognitive impairment*':ti,ab,kw OR 'delirium':ti,ab,kw OR 'dementia':ti,ab,kw OR 'mental deterioration*':ti,ab,kw OR 'overinclusion':ti,ab,kw OR 'response interference':ti,ab,kw |
| #5 | 'cognitive rehabilitation'/exp |
| #6 | 'brain training':ti,ab,kw OR 'cognitive rehabilitation':ti,ab,kw OR 'cognitive training':ti,ab,kw OR 'memory training':ti,ab,kw |
| #7 | (#1 OR #2) AND (#3 OR #4) AND (#5 OR #6) |

**4.Web of science**

| Search number | Query |
| --- | --- |
| #1 | "stroke* (Title) OR apoplex* (Title) OR ischaemic seizure (Title) OR brain attack (Title) OR brain blood flow disturbance (Title) OR brain insult* (Title) OR cerebral* (Title) OR cerebrovascular (Title) OR ischemic seizure (Title) OR brain accident (Title) OR Vascular Accident* (Title) OR Cerebrovascular accident (Title)" |
| #2 | "Cognitive Dysfunction (Title) OR cognitive defect (Title) OR Cognitive Decline (Title) OR cognition disorder* (Title) OR cognitive defect* (Title) OR amnestic (Title) OR cognitive deficit (Title) OR cognitive disability (Title) OR Cognitive Disorder* (Title) OR Cognitive Dysfunction* (Title) OR Cognitive Impairment* (Title) OR delirium (Title) OR dementia (Title) OR Mental Deterioration* (Title) OR overinclusion (Title) OR response interference (Title) " |
| #3 | "cognitive training (Title) OR cognitive rehabilitation (Title) OR brain training (Title) OR Memory Training (Title) " |

**5. The following table shows an example of a search strategy for Chinese databases (Sinomed).**

| Search number | Query |
| --- | --- |
| #1 | "stroke"[unweighted: extend] |
| #2 | ("cerebral apoplexy"[common field: intelligently] OR "cerebral arterial thrombosis"[common field: intelligently] OR "cerebral infraction"[common field: intelligently] OR "cerebral hemorrhage"[common field: intelligently] OR "hemorrhagic apoplexy"[common field: intelligently] OR "subarachnoid hemorrhage "[common field: intelligently]) |
| #3 | (("cerebral apoplexy"[common field: intelligently] OR "cerebral arterial thrombosis"[common field: intelligently] OR "cerebral infraction"[common field: intelligently] OR "cerebral hemorrhage"[common field: intelligently] OR "hemorrhagic apoplexy"[common field: intelligently] OR "subarachnoid hemorrhage "[common field: intelligently])) OR ("stroke"[unweighted: extend]) |
| #4 | "cognitive disorder"[unweighted: extend] |
| #5 | ("neurobehavioral disorders"[common field: intelligently] OR "cognitive impairment "[common field: intelligently] OR "cognitive deficit"[common field: intelligently] OR "cognitive disorders"[common field: intelligently]) |
| #6 | (("neurobehavioral disorders"[common field: intelligently] OR "cognitive impairment "[common field: intelligently] OR "cognitive deficit"[common field: intelligently] OR "cognitive disorders"[common field: intelligently])) OR ("cognitive disorder"[unweighted: extend]) |
| #7 | "cognitive training"[unweighted: extend] |
| #8 | ("cognitive function exercise"[common field: intelligently] OR "cognitive exercise"[common field: intelligently] OR "cognitive restructuring"[common field: intelligently] OR " cognitive function training"[common field: intelligently] OR " cognitive function restructuring "[common field: intelligently]) OR ("cognitive therapy"[common field: intelligently] OR "cognitive treatment"[common field: intelligently] OR "recognition therapy"[common field: intelligently]) |
| #9 | (("cognitive function exercise"[common field: intelligently] OR "cognitive exercise"[common field: intelligently] OR "cognitive restructuring"[common field: intelligently] OR " cognitive function training"[common field: intelligently] OR " cognitive function restructuring "[common field: intelligently]) OR ("cognitive therapy"[common field: intelligently] OR "cognitive treatment"[common field: intelligently] OR "recognition therapy"[common field: intelligently])) OR ("cognitive training"[unweighted: extend]) |
| #10 | ((("cerebral apoplexy"[common field: intelligently] OR "cerebral arterial thrombosis"[common field: intelligently] OR "cerebral infraction"[common field: intelligently] OR "cerebral hemorrhage"[common field: intelligently] OR "hemorrhagic apoplexy"[common field: intelligently] OR "subarachnoid hemorrhage "[common field: intelligently])) OR ("stroke"[unweighted: extend])) AND ((("neurobehavioral disorders"[common field: intelligently] OR "cognitive impairment "[common field: intelligently] OR "cognitive deficit"[common field: intelligently] OR "cognitive disorders"[common field: intelligently])) OR ("cognitive disorder"[unweighted: extend])) AND ((("cognitive function exercise"[common field: intelligently] OR "cognitive exercise"[common field: intelligently] OR "cognitive restructuring"[common field: intelligently] OR " cognitive function training"[common field: intelligently] OR " cognitive function restructuring "[common field: intelligently]) OR ("cognitive therapy"[common field: intelligently] OR "cognitive treatment"[common field: intelligently] OR "recognition therapy"[common field: intelligently])) OR ("cognitive training"[unweighted: extend])) |
